# The Chromenopyridine Scaffold: A Privileged Platform in Drug Design

**DOI:** 10.3390/molecules29133004

**Published:** 2024-06-25

**Authors:** Fábio Pedroso de Lima, Marta Costa, Ana Sousa, Maria Fernanda Proença

**Affiliations:** 1 Chemistry Centre, School of Sciences, University of Minho, Gualtar Campus, 4715-303 Braga, Portugal; fabioplima.conceicao@gmail.com (F.P.d.L.); id9570@alunos.uminho.pt (A.S.); 2 Centre for Textile Science and Technology (2C2T), University of Minho, Azurém Campus, 4800-058 Guimarães, Portugal; 3 Life and Health Sciences Research Institute (ICVS), University of Minho, Gualtar Campus, 4710-057 Braga, Portugal; martafcosta@med.uminho.pt; 4 ICVS/3B’s—PT Government Associate Laboratory, 4710-057 Braga/Guimarães, Portugal

**Keywords:** pyridine, chromene, chromenopyridine, synthesis, biological activity

## Abstract

The chromenopyridine scaffold represents an important class of heterocyclic compounds exhibiting a broad spectrum of biological properties. This review describes novel and efficient procedures for the synthesis of this scaffold. Herein, several methods were detailed and grouped according to their starting material (e.g., salicylaldehydes, chromones, chromanones and coumarins) and respective biological activity, when reported. This review highlights the potential of the reported synthetic strategies for preparing chromenopyridine derivatives with promising biological activity, paving the way for further developments in drug discovery.

## 1. Introduction

Drug discovery and development is a long and difficult process and medicinal chemists have been searching for inspiration in nature and synthetic compounds, with proven biological potential, to develop novel and significant molecules [1,2]. Indeed, a number of molecular scaffolds present in many natural and synthetic compounds have proven to be excellent promoters for hit/lead development [1,2,3,4,5]. Several “privileged structures”, an expression introduced by Evans et al. in 1988, are nowadays used as templates for the development of organic compounds with impacts in biology and medicine [6]. The use of these privileged scaffolds to construct disruptive compound libraries by enhancing structural diversity and physicochemical properties have ultimately led to highly potent and safe bioactive compounds.

**Figure 1:**
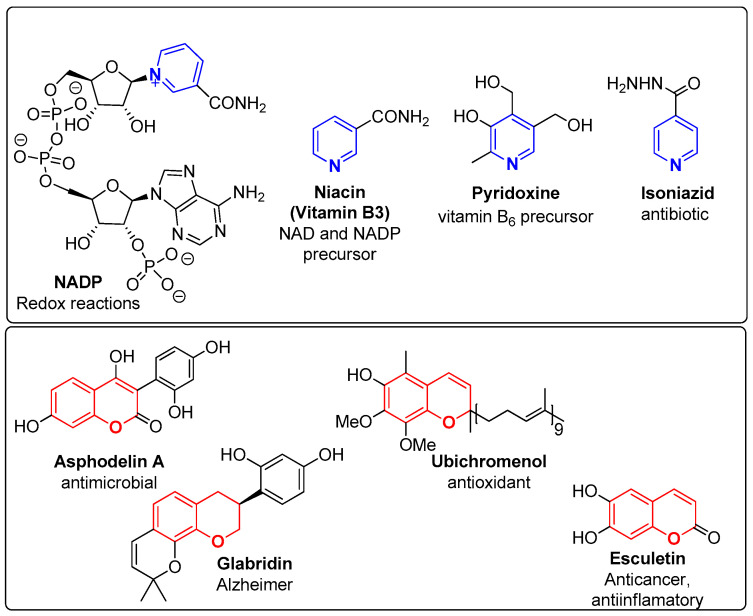
Chemical structure of natural and synthetic pyridines (Blue) and chromenes (Red) with diverse biological applications.

The pyridine unit can be found in natural and synthetic compounds, fused to other motifs, giving rise to compounds with interesting biological profiles [5,7,8,9,10] and is therefore considered an attractive scaffold [11,12]. This heterocyclic unit is present in a number of natural products such as nicotinamide adenine dinucleotide phosphate (NADP), vitamins B_3_ (niacin) and B_6_ (pyridoxine), in the alkaloid nicotine, as well as in several FDA-approved commercial drugs such as the antituberculotic isoniazid (Figure 1) [13]. Synthetic compounds incorporating the pyridine unit have also been developed and have presented diverse biological applications [2,3,4,5]. The synthesis of compounds bearing a 2,4-disubstituted pyridine ring as the central building block, presenting a potent inhibitory activity of the H_4_ histamine receptor (K_1_ < 10 nM), has been patented by Janssen Pharmaceutica [8,14]. Baraldi et al. [9] also reported the isolation of heterocyclic compounds bearing a pyridine ring fused with other aromatic moieties, such as pyrazolopyridines and imidazopyridines. These compounds proved to be potent antagonists of A_1_ and A_2A_ adenosine receptors, important receptors for the treatment of several diseases such as chronic obstructive pulmonary disease (COPD), asthma and others. More recently, the work of Martinez-Gualda et al. [10], involving new isothiazolo [3,4-*b*]pyridines, has led to the discovery of new selective inhibitors of the cyclin G-associated kinase (GAK), which is associated with the treatment of viral infections caused by hepatitis C, dengue, the Zika virus from the *Flaviviridae* family, the Ebola filovirus and others. Radwan et al. [15] recently reported the synthesis, molecular docking studies and antimicrobial activity of new tetrasubstituted pyridines. In relation to pyrazolo [3,4-*b*]pyridines, Jian et al. [16] described the synthesis of these compounds based on the combretastatin structure, which were tested for their antiproliferative and tubulin polymerization inhibitory activities and demonstrated their potential for further development as novel anticancer agents.

On the other hand, the chromene unit comprises heterocyclic compounds that incorporate a benzene ring fused to a pyran nucleus. Chromenes are present in several natural and synthetic compounds [17], and have been widely studied as therapeutic agents due to their low toxicity, especially reported for natural derivatives, and the versatility of their pharmacological applications [17]. Similarly to the pyridine moiety, the chromene scaffold appears in the literature as an important building block for the development of new bioactive compounds. Various chromene-based compounds have revealed interesting anticancer [18,19,20,21,22,23,24], antimicrobial [17,25,26,27], antibacterial [25,28,29] and antioxidant [27,30] activity, as well as other relevant biological applications, such as monoamine oxidase inhibitors [31]. Haiba et al. [23] detailed a series of benzo[*f*]chromenes with cytotoxic activity against HepG-2 and MCF-7 cancer cell lines. Abu El-Azm et al. [19] reported a series of benzo[*f*]chromene, chromeno [2,3-*d*]pyrimidine and chromenotriazolo [1,5-*c*]pyrimidine derivatives with relevant antiproliferative activity against MCF-7, HePG2 and WI-38 cancer cell lines, stating that the most probable mechanism of action may involve tubulin inhibition. Thahn et al. [29] described a click chemistry approach to synthesize 1*H*-1,2,3-triazole-tethered 4*H*-chromene-*D*-glucose conjugates with remarkable activity against Gram-positive and Gram-negative bacteria, which has also been tested with fungi. Well-known examples of natural chromene-based compounds are esculetin (anticancer agent), asphodelin A (antimicrobial agent), ubichromenol (antioxidant agent) and glabridin (Alzheimer disease) (Figure 1).

**Figure 2:**
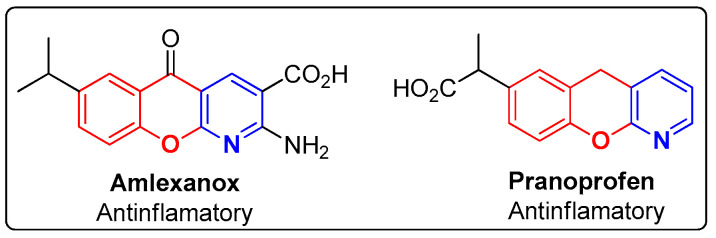
FDA-approved compounds based on the chromenopyridine moieties.

The design and synthesis of innovative compounds, combining the pyridine and chromene moieties, have been extensively reported in the literature, and these diversified structures have proven their potential as antinociceptive [32,33,34], alpha adrenergic antagonist [35], bronchodilator [36,37,38], antimicrobial [39] and antibiotic [40] agents, among others [41], including in FDA-approved drugs (Figure 2). Amlexanox, an antiallergic drug with clinical effectiveness against atopic diseases, allergic asthma and rhinitis, is a molecule with a chromenopyridine skeleton. Pranoprofen, a non-steroidal anti-inflammatory drug (NSAID) used in ophthalmology, constitutes another example of a chromenopyridine-based FDA approved drug [42].

This review is focused on these attractive scaffolds and presents a collection of synthetic methods for the preparation of chromenopyridines, including their potential as scaffolds for the development of novel bioactive compounds. A previously published review article, by Nunez-Vergara et al. [41], presents a good coverage of the synthesis of chromenopyridines up to 2011, which was mainly based on ANRORC-type mechanisms. This present work encompasses the latest updates on the involvement of chromenopyridines in the preparation of new compounds with biological activity, ranging from 2010 to 2023.

## 2. Synthesis and Biological Activity of Chromenopyridines

The different synthetic approaches to prepare this compound family were organized according to the starting material used. These include salicylaldehydes, chromones, chromanones, coumarins and miscellaneous/other sources. Whenever available, the biological activity of these chromenopyridines was reported, and also the reaction mechanism responsible for their formation.

### 2.1. Salicylaldehydes

The synthesis of chromenopyridines using salicylaldehydes as the starting material typically comprises a multi-component reaction (MCR), most commonly a three-component reaction, involving the appropriate salicylaldehyde, an active methylene compound and a nucleophile, under base catalysis. The presence of the nucleophile is paramount, as it promotes the intramolecular cyclization of the Knoevenagel adduct formed from the reaction between the active methylene compound and the salicylaldehyde into the final chromenopyridine scaffold.

**Scheme 1:**
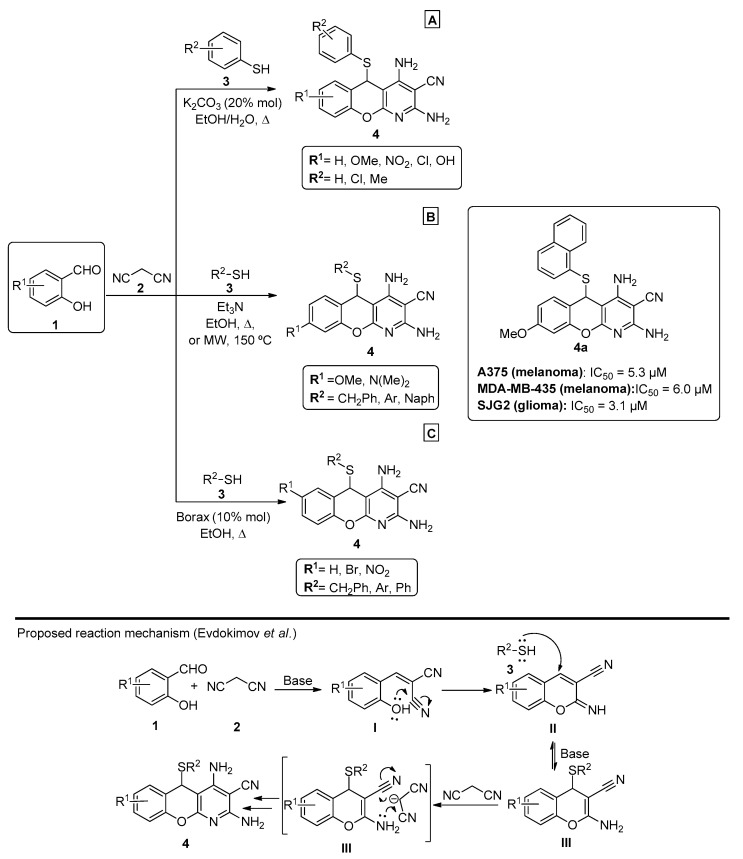
Preparation of chromenopyridines **4** with the anticancer potential (IC_50_) of compound **4a** highlighted and the reaction mechanism proposed by Evdokimov et al. (adapted from Refs. [12,43,44,45,46]). (**A**)—Mishra et al.; (**B**)—Banerjee et al.; (**C**)—Molla et al.

Regarding the use of malononitrile and its dimer, a 2011 publication by Mishra et al. [43] proposed an improvement to the method previously reported by Evdokimov et al. in 2006 and 2007 [12,44]. In this approach, a one-pot three-component reaction was explored using salicylaldehydes **1**, malononitrile **2** and different thiols **3**, in EtOH/H_2_O and under K_2_CO_3_ (20% mol) catalysis. In their work, the authors compared different bases that were previously reported for pyridine synthesis, including those evaluated in Evdokimov’s study, and concluded that K_2_CO_3_ afforded better reaction yields (up to 92%). Several chromenopyridines **4** were prepared in good-to-high yields (63–92%), under reflux in EtOH/H_2_O (Scheme 1A). A similar work was later reported by Banerjee et al. [45] (Scheme 1B), using triethylamine and either refluxing ethanol (88–90% yield) or microwave irradiation at 150 °C (30–45% yield). The anticancer potential of the compounds was tested for a series of melanoma and glioma cell lines, from which compound **4a** (R^1^ = OMe, R^2^ = Nap) was highlighted (Scheme 1B). A more recent publication by Molla et al. [46] showed good-to-high yields (85–91%) of chromenopyridine **4** when the reaction was performed using borax (10% mol) as a catalyst (Scheme 1C).

**Scheme 2:**
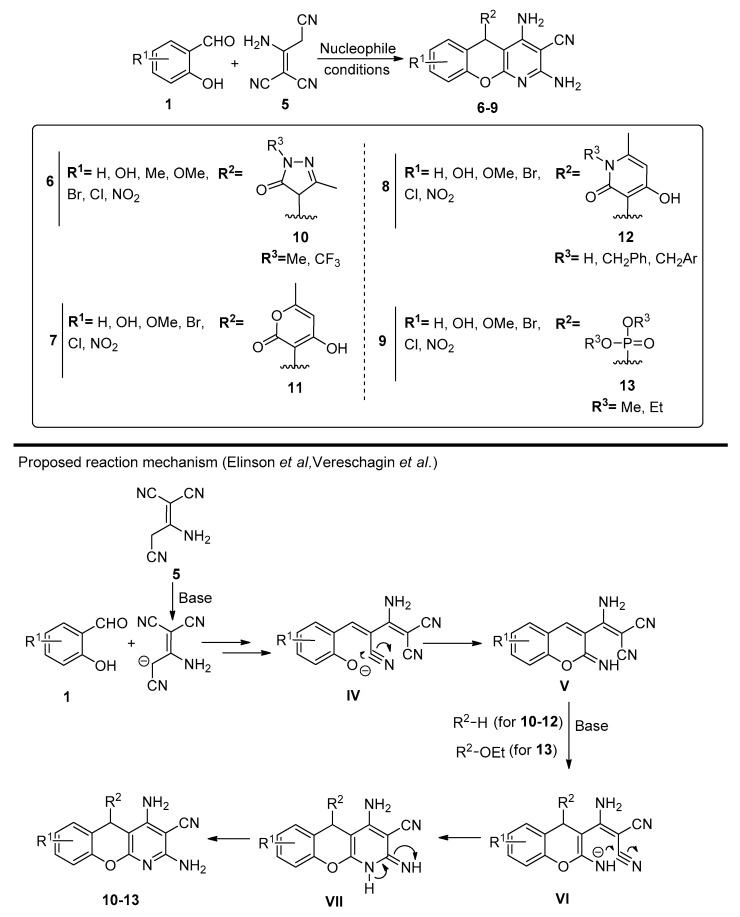
Preparation of chromenopyridines **6-9** and reaction mechanism proposed by the authors (adapted from Refs. [47,48,49,50,51,52,53,54,55,56]). Reaction conditions: synthesis of **6**: **10**, Et_3_N (10% mol), PrOH or MeCN; synthesis of **7**: **11**, EtOH/pyridine (3:1), 80 °C; synthesis of **8**: **12**, EtOH/pyridine (3:1), 80 °C; synthesis of **9**: **13**, morpholine (10% mol), MeCN, reflux (adapted from Refs. [46,47,48,49,50,51,52,53,54,55,56]).

Several studies by Vereschagin et al. and Elinson et al. [47,48,49,50,51,52,53,54,55,56] report the preparation of new chromenopyridines from a three-component reaction involving the dimer of malononitrile, 2-aminoprop-1-ene-1,1,3-tricarbonitrile. This dimer can be previously synthesized or generated in situ [48], and was reacted with salicylaldehydes **1** by Knoevenagel condensation. Formation of the 3-substituted 2-iminochromene, followed by intramolecular cyclization and Michael addition of a nucleophile to the chromene ring generated the chromenopyridine scaffold (Scheme 2). These reactions are characterized by their simplicity, facile work-up methods and high atom economy. Over the last 12 years, several studies have used this methodology to prepare different substituted chromenopyridines, mainly varying the reaction conditions and the nucleophile used, usually under basic conditions. Elinson et al. published a selection of articles on this subject [50,51,52,53,54,56], using different nucleophiles and reaction conditions. 

The synthesis of chromenopyridines **6** was performed by reaction of salycilaldehydes **1**, 2-aminoprop-1-ene-1,1,3-tricarbonitrile **5** and pyrazoline-5-ones **10**, under triethylamine catalysis (10% mol), in either propanol or acetonitrile, in high-to-almost quantitative yields [50,51]. Chromenopyridines **7** and **8** were also prepared in good yields (49–85% for **7**, up to 97% for **8**) by reacting salicylaldehydes **1**, 2-aminoprop-1-ene-1,1,3-tricarbonitrile **5** and 4-hydroxy-6-methyl-2*H*-pyran-2-one **11** or substituted 4-hydroxy-6-methylpyridin-2(1*H*)-ones **12**, in a mixture of EtOH/pyridine 3:1 at 80 °C [53]. Chromenopyridines **9** [54,56] were prepared in good-to-high yields (56–86%) by a similar approach, using trialkyl phosphites **13** as nucleophiles, with morpholine (10% mol) catalysis and under reflux conditions in acetonitrile.

**Scheme 3:**
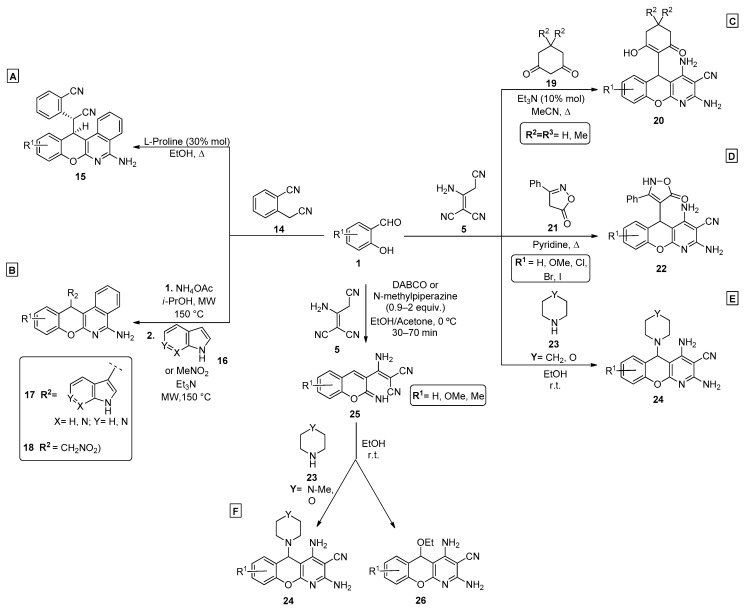
Preparation of chromenopyridines **15**, **17**, **18**, **20**, **22**, **24**, and **26**. (**A**,**B**)—Festa et al.; (**C**,**D**)—Vereshchagin et al.; (**E**)—Shaabani et al.; (**F**)—Lopes et al. (adapted from Refs. [47,48,49,57,58,59,60]).

The use of homophtalonitrile **14** instead of malononitrile or derivates, as a precursor in the synthesis of chromenopyridines, has also been studied in the past years. Two recent publications [57,58] describe the experimental conditions to prepare chromenopyridines **15**, **17** and **18** by a multicomponent reaction involving **14**, salicylaldehydes **1** and different nucleophiles (Scheme 3A,B). The proposed methodologies are conceptually similar to those involving other active methylene compounds and are also based on the formation of an adduct from Knoevenagel condensation, followed by a tandem intramolecular cyclization promoted by Michael addition of a nucleophile, leading to the formation of the chromenopyridine scaffold. For the synthesis of compound **15**, salicylaldehydes **1** and two equivalents of homophtalonitrile **14** were refluxed in ethanol, under *L*-Proline (30% mol) catalysis. The authors suggest that *L*-proline may aid in the Michael addition step of the second homophtalonitrile unit, acting as a base (Scheme 3A). For the synthesis of compounds **17** and **18**, the best results were obtained by a two-step process where homophtalonitrile **14** and the appropriate salicylaldehyde **1** were reacted at 150 °C for 10 min, under microwave irradiation and ammonium acetate catalysis. Addition of an excess of the appropriate nucleophile (**16**) and one equivalent of triethylamine and heating at 150 °C for a further 10 min period, under microwave irradiation, led to product **17** (Scheme 3B). 

Vereshchagin et al. [48,49], in collaboration with Elinson’s group, developed similar procedures to prepare chromenopyridines from malononitrile dimer in a one-pot three-component reaction. One of the methods [49] combined salicylaldehydes **1**, aminoprop-1-ene-1,1,3-tricarbonitrile **5** and 1,3-cyclohexanediones **19** in acetonitrile and under reflux conditions, with a catalytic amount of triethylamine, to prepare chromenopyridines **20** in good-to-high yields (59–88%) (Scheme 3C). Molecular docking studies of compounds **20** with mitogen-activated protein kinases 1 and 2 binding pockets showed favorable binding free energy values, which highlight their biological potential for the development of anticancer agents. In another work [48], the preparation of chromenopyridines **20** involved the use of malononitrile and formation of the dimer was considered to occur in situ under the experimental conditions that were used. This study showed that the best results were obtained when using triethylamine (10% mol) and acetonitrile, under reflux, leading to compound **20** in 53–87% yields. The same author also presented a method [47] to prepare chromenopyridines **22**, by performing the reaction with **5** and 3-phenylisoxazol-5(4*H*)-one **21** in pyridine and under reflux, where pyridine behaved both as solvent and catalyst (Scheme 3D). The final products were obtained in 65–74% yields. A different work, by Shaabani et al. [59], described a multicomponent reaction involving salicylaldehydes **1**, aminoprop-1-ene-1,1,3-tricarbonitrile **5** and secondary cyclic amines **23** (Scheme 3E). With this method, piperidine and morpholine were incorporated into the chromenopyridine scaffold, by reacting as nucleophiles in the intramolecular cyclization steps, in a reaction performed in ethanol, at room temperature, affording chromenopyridines **24** in high yields (80–94%).

A similar work was published by Lopes et al. [60], where different nucleophiles were incorporated in the chromenopyridine moiety. The procedure involved a two-step reaction that starts from 2-aminoprop-1-ene-1,1,3-tricarbonitrile **5** and salicylaldehydes **1**, from which compound **25** was isolated as a pure product in 71–84% yields only when the reaction was performed at 0 °C. Intramolecular cyclization involving either *N*-methylpiperazine, morpholine, or ethanol, acting as nucleophiles, resulted in the final chromenopyridine scaffold (compounds **24** and **26**, Scheme 3F). A selection of these compounds were tested for their anticancer activity against breast cancer cell lines where an interesting anticancer profile for the luminal breast subtype was identified for derivative **24** (R^1^ = Me or OMe, Y = N-Me). These compounds presented the capacity to inhibit cell proliferation (low micromolar range) and induced cell cycle arrest, promoting apoptosis and microtubule destabilization, having also demonstrated a good safety profile in the in vivo *C. elegans* toxicity assay.

**Scheme 4:**
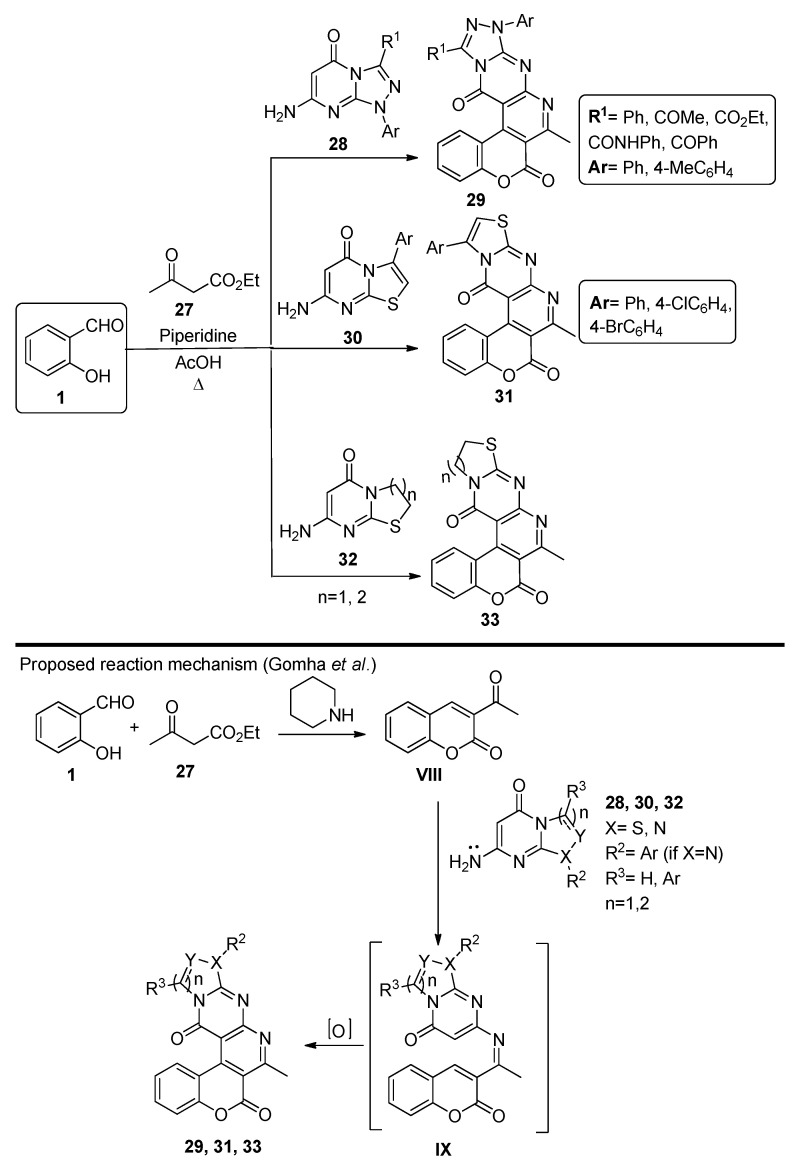
Preparation of chromenopyridines **29**, **31** and **33** and reaction mechanism proposed by Gomha et al. (adapted from Ref. [61]).

In 2014, Gomha et al. [61] developed a three-component reaction involving salicylaldehyde, ethyl acetoacetate **27** and compounds **28**, **30** and **32**, to originate chromenopyridines **29**, **31** and **33**, respectively (Scheme 4). The reaction occurred under reflux conditions, using acetic acid as solvent and with piperidine catalysis, and afforded compounds **29**, **31** and **33** in 66–77, 73–84, and 73–78% isolated yields, respectively. The authors proposed that the reaction starts with the base-catalyzed condensation of salicylaldehyde **1** with ethyl acetoacetate **27**, followed by intramolecular cyclization involving the ester group and elimination of ethanol. Addition of the heterocyclic enamine afforded the chromenopyridines **29**, **31**, or **33**. 

**Scheme 5:**
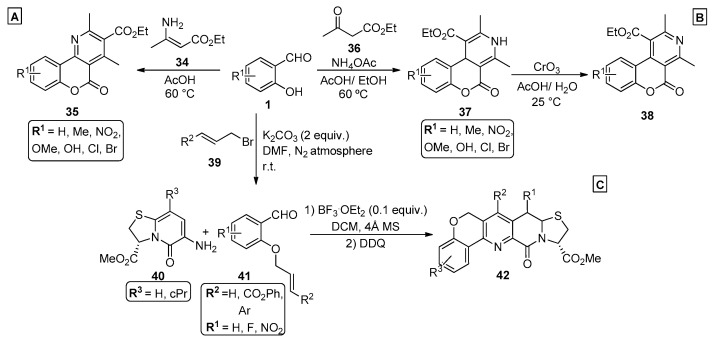
Preparation of chromenopyridines **35**, **37**, **38** and **42** (adapted from Refs. [62,63]). (**A**,**B**)—Navarrete-Encina et al.; (**C**)—Adolfsson et al.

In the typical Hantzsch pyridine synthesis, a one-pot three-component reaction between an aldehyde, two molar equivalents of a β-ketoester and a nitrogen donor occurs to generate the pyridine ring. This procedure was directly applied to the synthesis of chromenopyridines by using salicylaldehydes. In their work, Navarrete-Encina et al. demonstrated that chromeno [3,4-*c*]pyridine-3-carboxylates **35** and chromeno [4,3-*b*]pyridine-3-carboxylates **37** could be synthetized from the reaction of salycilaldehydes **1** with either two equivalents of ethylaminocrotonate **34** or three equivalents of ethyl acetoacetate **36**, respectively (Scheme 5A,B) [62]. The authors suggested that the reaction was initiated by the formation of the benzopyran ring by condensation of **36** with the aldehyde. The formation of the pyridine ring started by a 1,4-addition of either the Cα or the amino group of ethylaminocrotonate **34**, to the benzopyran C4 atom. This determined the regioselectivity of the general process that is mainly favored by the reaction conditions used. The use of ethylaminocrotonate **34** in a reaction performed at 60 °C, and using acetic acid as solvent, led to the formation of regioisomer **35** (Scheme 5A). Reacting salicylaldehyde **1** with ammonium acetate and ethyl acetoacetate **36** under reflux, in a 1:1 mixture of acetic acid/ethanol, led to the formation of regioisomer **37**, which was further oxidized to the final chromenopyridine moiety (**38**, Scheme 5B).

The Povarov reaction [63] is a useful tool to prepare nitrogen-containing six-membered rings through a cycloaddition reaction. This method has been recently used by Adolfsson et al. [64] to generate the chromenopyridine-fused 2-pyridones **42** by the reaction of amino-2-pyridones **40** and *O*-alkylated salicylaldehydes **41** under BF_3_·OEt_2_ catalysis, followed by DDQ oxidation (Scheme 5C). Using this methodology, 11 derivatives of **42** (R^1^ = Ar, R^2^ = H, cPr) were prepared in good-to-high yields (53–93%) when the *O*-alkylated salicylaldehyde **41** contained a terminal allyl moiety, while 5 other derivatives of **42** (R^1^ = H, R^2^ = H, cPr) were isolated in low-to-moderate yields (13–54%) from the *O*-alkylated salicylaldehyde **41**.

### 2.2. Chromones

Chromones, and in particular substituted chromones, have been widely used as precursors for the chromenopyridine scaffold and most of the reactions proceed through an ANRORC-type mechanism [43]. The first reaction step typically involves Michael addition of an active methylene compound to either position 2 or position 4 of the chromone derivative. This is followed by ring opening, bond rotation and ring closure to generate the chromene ring. For the formation of the chromenopyridine ring, the chromone precursor is typically substituted with a nitrile group at position 3 that will afford the nitrogen in the pyridine moiety. 

**Scheme 6:**
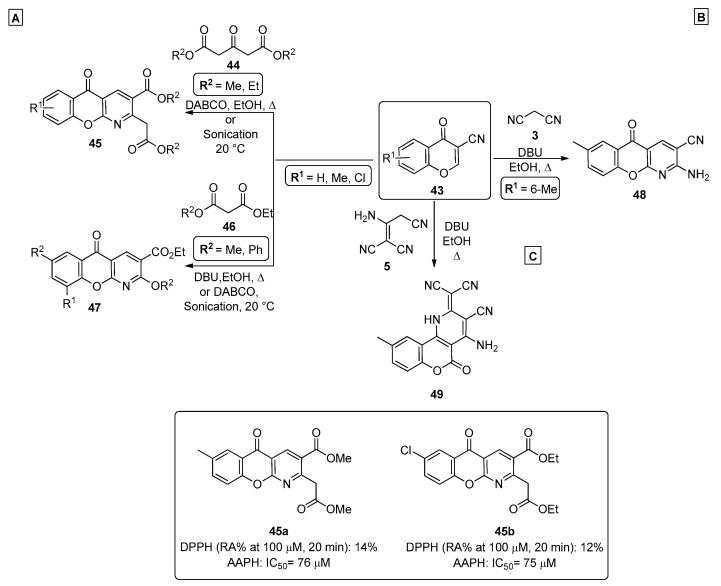
Preparation of chromenopyridines **45**, **47**, **48** and **49** and antioxidant activity (IC_50_, RA = reducing agent) of compounds **45a** and **45b** highlighted (adapted from Refs. [65,66,67]). (**A**)—Dimitriadou et al.; (**B**,**C**)—Ibrahim et al.

In 2014, Dimitriadou et al. [65] prepared a series of chromenopyridine derivatives **45** and **47**, by reacting 3-cyanochromone **43** with dimethyl- or diethyl 3-oxopentanedioates **44** or 1,3-dicarbonyl compounds **46** in the presence of base catalysis (DABCO or DBU) (Scheme 6A). Products **45** and **47** were prepared from chromone **43** under reflux in ethanol in overall moderate-to-good yields (47–54%). For these products, an additional method using ultrasound sonication and DABCO, at 20 °C, was also reported, leading to higher reaction yields (70–78%). A batch of compounds **45** and **47** was prepared and tested for their antioxidant activity, where their capacity to interact with 1,1-diphenylpicrylhydrazyl (DPPH) and with the azo compound 2,2″-azobis(2-amidinopropane) dihydrochloride (APPH) was measured. The results were compared with a standard, nordihydroguairetic acid (NDGA). The best results were obtained for derivatives **45a** (R^1^ = 7-CH_3_, R^2^ = CH_3_) and **45b** (R^1^ = 7-Cl, R^2^ = CH_2_CH_3_). 

Ibrahim et al. reported the synthesis of diverse chromenopyridines by a cascade process starting from a 3-cyanochromone **43** and involving ring opening of the pyran ring initiated by nucleophilic attack of the malononitrile anion (Scheme 6B) [66]. This is followed by intramolecular cyclization, with nucleophilic attack of the phenolate oxygen to the nitrile group in position 3, generating an imine that cyclized through nucleophilic attack to another nitrile group. The reaction was performed in ethanol, under reflux, using DBU as a base, leading to chromenopyridine **48**. Ibrahim et al. also reported the preparation of new chromenopyridine derivatives **49** by reacting 6-methylchromone-3-carbonitrile **43** with the malononitrile dimer **5** [67]. The reaction followed an ANRORC-type mechanism, where the nitrile group was involved in the formation of the pyridine ring, fused to C3 and C4 carbons of the newly formed coumarin ring (Scheme 6C).

**Scheme 7:**
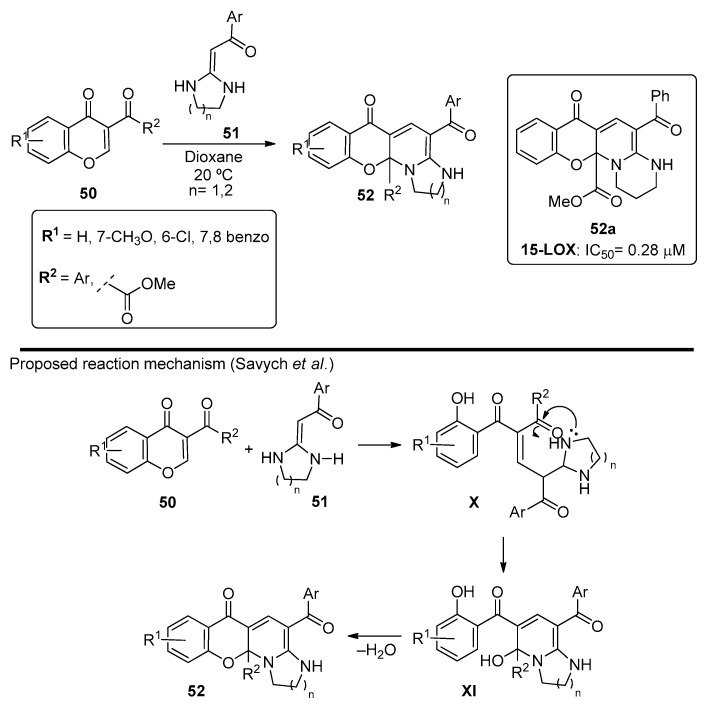
Preparation of chromenopyridines **52** with the antioxidant activity (IC_50_) of compound **52a** highlighted and reaction mechanism proposed by Savych et al. (adapted from Ref. [68]).

The synthesis of tetracyclic chromonopyridines via Michael addition using heterocyclic ketene aminals as nucleophiles was also reported by Savych et al. [68]. The chromonopyridine structure was generated from nucleophilic attack of the aminal **51** to chromones **50**. Ring opening of the pyran ring followed by ring closure to the ketone in the 3-position ultimately led to chromenopyridines **52** (Scheme 7) in good-to-high yields (75–92%). These compounds were studied by DFT and molecular docking calculations, and the structure was confirmed by X-ray crystallography. The anti-inflammatory properties were also assessed through 15-Lipoxigenase (15-LOX) inhibition studies, and the best results (56-fold higher than the standard, quercetin) were obtained for R^1^ = H, R^2^ = COOCH_3_, Ar = Ph, and n = 2 (Scheme 7, compound **52a**). 

**Scheme 8:**
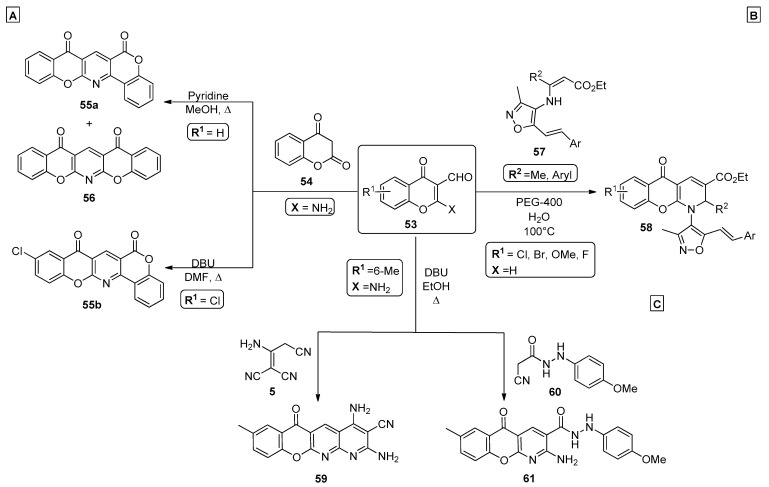
Preparation of chromenopyridines **55**, **56**, **58**, **59** and **61** (adapted from Refs. [67,69,70,71]). (**A**)—Siddiqui et al., Ibrahim et al.; (**B**)—Pondurii et al.; (**C**)—Ibrahim et al.

A simple and convenient procedure for the preparation of chromenopyridine derivatives involves the formation of the pyridine ring either by reaction of a chromone with a nucleophile incorporating an amino group or by reaction of a chromone incorporating an amino group with a nucleophile. For example, Siddiqui et al. [69] synthesized two new chromenopyridines (mixture of **55a** and **56**) in a 1:2 ratio by reaction of 2-amino-4-oxo-4*H*-chromene-3-carbaldehyde **53** with chroman-2,4-dione **54**, in refluxing methanol and in the presence of a catalytic amount of pyridine (Scheme 8A). In 2016, Ibrahim et al. [70] developed a simple condensation procedure to afford pentacyclic chromenopyridines **55b** from the DBU-catalyzed reaction of 4*H*-chromene-3-carbaldehyde **53** and chroman-2,4-dione **54**. By performing the reaction in DMF and under reflux conditions, compound **55b** was obtained in a 54% yield (Scheme 8A). Further optical studies proceeded, and the results indicated that this scaffold could be tuned to enhance its photochemical properties, to be used as a photodetector.

Ponduri et al. explored the reactivity of isoxazolyl enamino esters **57** with 3-formylchromones **53** to generate new chromenopyridine-based compounds **58** (Scheme 8B) [71]. Optimization of the reaction conditions showed that water was the best solvent, while the use of PEG-400 (10% mol) catalysis at 100 °C was also optimal. This procedure allowed the preparation of isoxazolyl-substituted chromenopyridines **58** in good-to-high yields (75–92%). Mechanistically, it was suggested that PEG catalyzes the condensation of isoxazolyl enamino esters and 3-formylchromones by facilitating the nucleophilic attack between the enamine nucleophile **57** and the aldehyde group of **53**. After condensation, a 6π-electrocyclization occurs to afford the pyridine ring of **58**. Alternatively, the chromenopyridine can be formed by condensation of a substituted 2-aminochromone with a nucleophile, followed by an intramolecular cyclization reaction. In these reactions, the nitrogen of the pyridine ring can come from the chromone or chromane moiety, typically in the form of an amine group, or from the nucleophile that is condensed with the chromene ring. Ibrahim et al. also reported the preparation of new chromenopyridine derivatives **59** by reacting 4*H*-chromone **53** with the malononitrile dimer **5** (Scheme 8C). Compound **53** was also reacted with 2-cyano-acetohydrazide **60** in the presence of DBU and reflux in ethanol, to afford chromenopyridines **61** (Scheme 8C) [67].

**Scheme 9:**
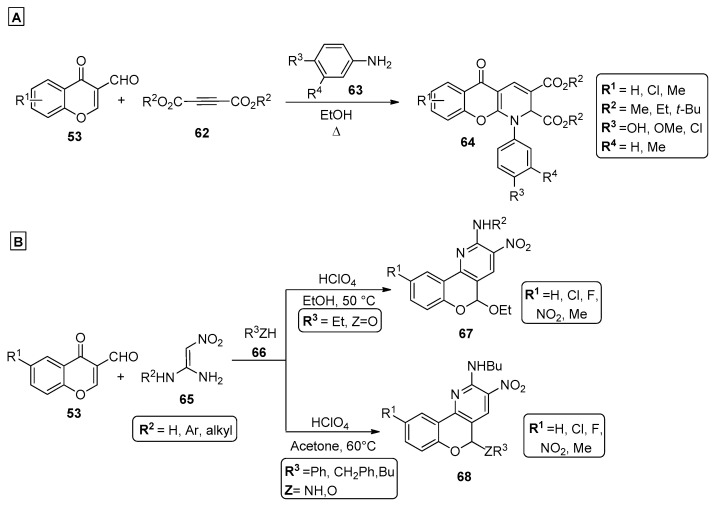
Preparation of chromenopyridines **64**, **67** and **68** (adapted from Refs. [72,73]). (**A**) Dolatkhah et al.; (**B**) Zhang et al.

In a methodology reported by Dolatkhah et al. [72], benzochromonodiazocines were reported as the only product of the reaction of 3-formylchromones **53**, dialkyl acetylenedicarboxylates **62** and aromatic amines **63** under reflux in ethanol (Scheme 9A). Through a convenient and simple reaction, also with simple work-up protocols, chromenopyridines **64** were obtained in high yields (70–86%). Although no mechanistic studies were discussed in this work, it is suggested that the most probable mechanism involves a Michael addition of amine **63** to chromone **53**, followed by condensation of the resulting intermediate with **62** and subsequent cyclization into the final product **64.** Chromenopyridines incorporating a nitro group could be synthesized in moderate-to-high yields via a perchloric acid-catalyzed three-component reaction (Scheme 9B) [73]. This starts from the reaction of 3-formylchromenes **53**, 1,1-enediamines **65** and nucleophiles **66**, under mild conditions (ethanol or acetone, 50–60 °C). After optimizing the reaction conditions, the authors highlight acetone as the best solvent when ethanol is not used as a nucleophile, and perchloric acid as the best catalyst among several other acids used (the product was formed with a 81% yield). It was also reported that using temperatures higher than 50 °C did not favor the chromenopyridine formation. It was suggested that the reaction starts by Michael addition of **65** to the aldehyde of **53**, generating an intermediate with high degree of regioselectivity. The reaction proceeds through intramolecular cyclization, generating the 6-membered ring, which after imine-enamine tautomerization, acid-catalyzed water elimination and oxidation, leads to the chromenopyridine moiety of compounds **67** or **68**, depending on the experimental conditions used.

**Scheme 10:**
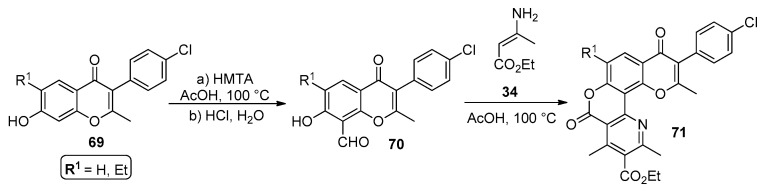
Preparation of chromenopyridines **71** as proposed by Lozinski et al. (adapted from Ref. [74]).

In the work performed by Lozinski et al. [74], a modified version of the Hantzsch synthesis afforded chromenopyridines **71** in good-to-moderate yields (43–69%). The procedure starts with the reaction of compound **69** with hexamethylenetetramine (HMTA) in acetic acid, followed by water addition and acidification with HCl, leading to **70** in a high yield (80–90%). Then, the modified Hantzsch reaction of **70** with an excess of 3-aminocrotonate **34** afforded chromenopyridines **71**. According to the authors, a predominance of C–N addition over the C–C addition pathway led to **71**, which was explained by the electronic effects within the 8-formyl-7-hydroxychromone scaffold **70** (Scheme 10).

### 2.3. Chromanones

**Scheme 11:**
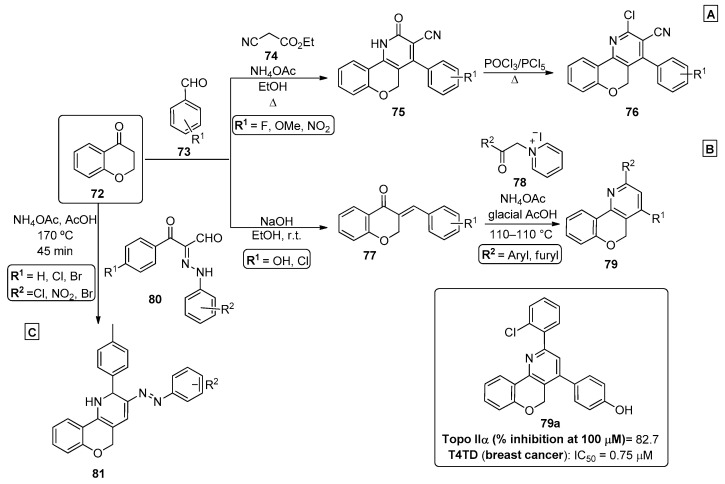
Preparation of chromenopyridines **76**, **79** and **81** with the biological activity of compound **79a** highlighted (adapted from Refs. [75,76,77,78,79,80,81]). (**A**) Ali et al.; (**B**) Thapa et al.; (**C**) Behbehani et al.

The use of chromanones as starting material for the preparation of chromenopyridines was also reported in the literature, and they present a similar chemical reactivity to that observed for chromones and substituted chromones. For example, to prepare new chromenopyridine derivatives **76**, a four-component reaction was developed [75]. Chroman-4-one **72** reacted with aromatic aldehydes **73**, ethyl cyanoacetate **74** and ammonium acetate under reflux in ethanol, affording compounds **75** in 65–82% isolated yield (Scheme 11A). These products were converted into the chlorinated chromenopyridines **76** (69–79% yields) by reaction with phosphonyl chloride, in a POCl_3_/PCl_5_ mixture under reflux conditions. 

In their work, Thapa et al. [76,77] developed a new multicomponent reaction to prepare α-terthiophene bioisosters based on the chromenopyridine moiety, in order to enhance the rigidity of the scaffold predicted to improve its biological activity (Scheme 11B). Aldol condensation of chroman-4-one **77** with diverse aldehydes **73** under base conditions led to chromanones **77** that evolved to chromenopyridines **79** by heating at 100–110 °C with diverse 2-oxoalkylpyridinium iodides **78** in the presence of ammonium acetate and glacial acetic acid. The resulting products **79** were tested for their activity as topoisomerase (Topo) I and II inhibitors, as well as for their cytotoxicity against several human cancer cell lines. The authors considered the isolated compounds as novel chromenopyridine-based Topo I and II inhibitors [78,79,80]. Compounds **79**, with phenyl or thienyl substituents, were among the most relevant in terms of selectivity against Topo II. Moreover, this new batch of chromenopyridine derivatives showed interesting and improved results in various cancer cell lines, including breast cancer cell lines T47D. The selectivity towards breast cancer cell lines versus non-tumorigenic breast cell line MCF-10A was noteworthy, with some compounds having an 18-fold enhanced inhibitory capacity when compared with the reference compound. Further SAR studies proceeded with compound **79a**, highlighted for its Topo I and IIα inhibitory capacity, as well as for its potent and selective antiproliferative activity via Topo Iiα inhibition (Scheme 11B) [80]. In 2021, Behbehani et al. [81] proposed a green protocol for the development of thiochromeno [4,3-*b*]pyridine and chromeno [4,3-*b*]pyridine derivatives, in which a sealed Q-tube reactor was used to perform the reaction under high pressure. In this procedure, a three-component reaction between chroman-4-one **72**, arylhydrazonals **80** and ammonium acetate, in acetic acid and at 170 °C, led to chromenopyridines **81** isolated in 84–92% yields (Scheme 11C).

**Scheme 12:**
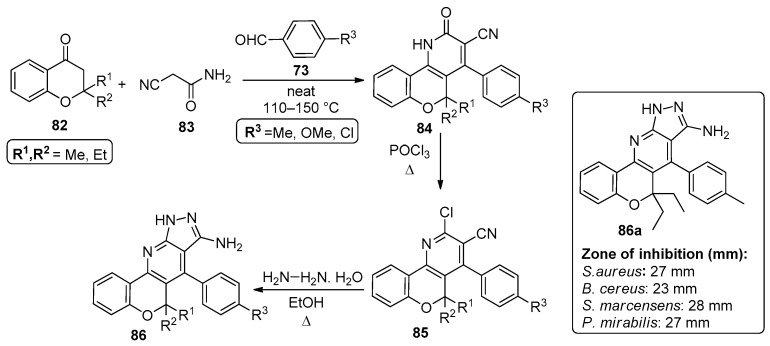
Preparation of chromenopyridines **85** and **86** with the antibacterial activity (zone of inhibition, mm) of compound **86a** highlighted (adapted from Ref. [82]).

In 2014, El-Essawy’s group published a one-pot three-component reaction involving 2,2-dialkylchroman-4-ones **82**, 2-cyanoacetamide **83** and 4-substituted aldehydes **73**, in a solventless reaction performed at 110–150 °C, leading to chromenopyridines **85** in 62–73% yields (Scheme 12) [82]. The synthesis of compounds **84** had been previously reported [83] and in the more recent work, compounds **85** were prepared through POCl_3_ chlorination of **84** and used in the synthesis of **86** by reaction with hydrazine monohydrate under reflux in ethanol. Chromenopyridines **84-86** were tested for their antibacterial activity against *S. aureus* (SA), *B. cereus* (BC), *S. marcensens* (SM) and *P. mirabilis* (PM), revealing an interesting antibacterial profile that was transversal to all the tested chromenopyridine subfamilies. Several compounds revealed relevant activity against more than one bacterial strain. Among the tested compounds, **86a** (R^1^ = R^2^ = Et) was highlighted for the diameter of its inhibition zone and announced as a promising drug candidate when compared to the standard ampicillin (Scheme 12).

### 2.4. Coumarins

**Scheme 13:**
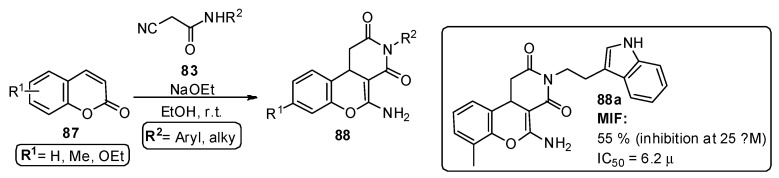
Preparation of chromenopyridines **88**, with the respective biological activity (MIF inhibition at 25 µM and respective IC_50_) highlighted for compound **88a** (adapted from Ref. [84]).

Kok et al. [84] reported the development of new macrophage migration inhibitory factor (MIF) inhibitors, referenced as an important target in the inflammatory process, signaling tumor cell growth in inflammatory disease models. In this study, 57 amino-2*H*-chromenopyridine-dione derivatives **88** were prepared from the reaction of cyanoacetamides **83** and substituted coumarins **87**, under mild conditions and in the presence of sodium ethoxide, with good and very good yields (Scheme 13). Most of the tested compounds showed MIF tautomerase inhibitory capacity, with IC_50_ in the low micromolar range, particularly compound **88a** (Scheme 13). 

**Scheme 14:**
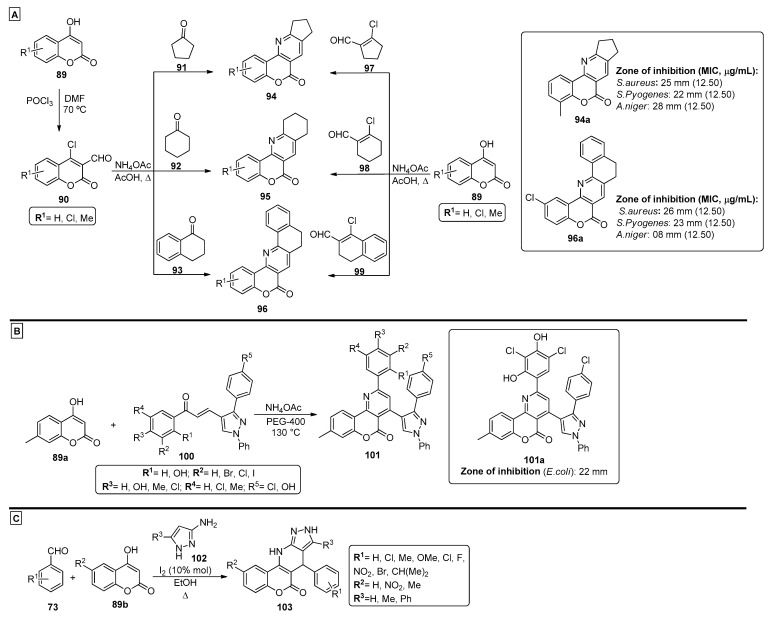
Preparation of chromenopyridines **94**–**96**, **101** and **103**, with the respective biological activity (zone of inhibition, mm and MIC, µg/mL) highlighted for compounds **94a**, **96a** and **101a** (adapted from Refs. [40,85,86]). (**A**)—Patel et al.; (**B**)—Dawane et al.; (**C**)—Pal et al.

Patel et al. [85] proposed a methodology for the preparation of novel chromenopyridines **94**–**96** from substituted 4-hydroxy-2*H*-chromen-2-ones **89** (Scheme 14A). The synthetic route starts by converting compounds **89** into the formylated and chlorinated coumarins **90** using POCl_3_ in DMF, followed by reaction with cyclic ketones **91-93** and ammonium acetate. Alternatively, the direct reaction of compounds **89** with cyclic chloroformyl compounds **97**–**99** and ammonium acetate was also used. The products were evaluated for their antimicrobial activity, and all tested compounds presented better inhibitory activity for Gram-positive bacteria than for Gram-negative bacteria. For the tested compounds, products **94a** and **96a** were among the most active structures, with interesting minimum inhibitory concentrations (MIC) and zone of inhibition results against *S. aureus* (SA), *S. pyogenes* (SP) and *A. niger* (AN) (Scheme 14A). Dawane et al. also proposed the formation of chromenopyridines **101** by a PEG-400-catalyzed MCR [40]. In this case, 4-hydroxy-7-methyl-coumarin **89a** was reacted with different chalcones **100** and ammonium acetate, at 130 °C, using PEG-400 as solvent (Scheme 14B). This method produced diarylsubstituted chromenopyridines **101** in 81–89% yields, in a process that allowed a facile separation of the final product and reusage of the PEG-400 solvent. The proposed mechanism for the formation of compounds **101** involved the in situ generation of 4-amino-7-methylcoumarin and subsequent Michael addition of the enamine unit to **100**, finishing with a ring closing step to form the pyridine ring. These compounds were tested against an array of microorganisms (e.g., *E. coli* and *A. flavus*), and several compounds demonstrated good results in one or more tested bacteria strains. A promising germination inhibition in several tested fungi was also reported. Compound **101a** (R^1^ = R^3^ = OH, R^2^ = R^4^ = R^5^ = Cl) showed significant inhibition of E. coli when compared with penicillin and was able to reduce or inhibit growth in all fungi tested. The use of iodine as a catalyst was also explored by Pal et al. [86]. In this work, the iodine-catalyzed reaction between aromatic aldehydes **73**, 4-hydroxycoumarins **89b** and different amino pyrazole derivatives **102** was studied. Optimization studies showed that the use of 10% mol of I_2_ as catalyst, under reflux in ethanol, were the best conditions for this reaction (Scheme 14C). Chromenopyridines **103** were prepared by a green and convenient method, with environmentally friendly catalysts and solvents and no chromatographic purification methods required. The authors also suggested that compounds **103** could be prepared from a four-component one-pot reaction, where the amino pyrazoles **102** were generated in situ from the reaction of benzoylacetonitrile and hydrazine monohydrate. The most likely mechanism to originate compounds **103** involves a Knoevenagel condensation between aldehydes **73** and 4-hydroxycoumarins **89b**, followed by Michael addition of aminopyrazole **102** to the newly formed adduct, which then undergoes a tandem intramolecular cyclization reaction. The authors also suggested that iodine acts as catalyst by coordinating to the carbonyl moieties, enhancing their electrophilicity, which facilitates nucleophilic attack in the Knoevenagel condensation, Michael addition and intramolecular condensation steps.

**Scheme 15:**
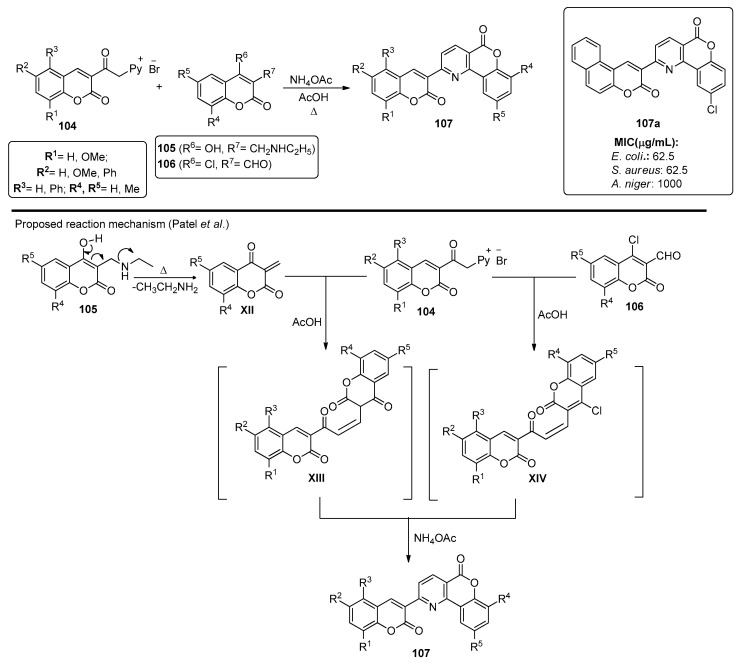
Preparation of chromenopyridines **107**, with the biological activity (MIC, µg/mL) highlighted for compound **107a** adapted from Ref. [87]).

Patel et al. also presented a method for preparing bischromeno structures **107**, starting from chromenylpyridinium salts **104** [87]. Compounds **104** were either reacted with chromen-2-ones **105** or chromene-3-carbaldehydes **106**, in the presence of ammonium acetate and reflux in acetic acid, to afford chromenopyridines **107**. The reaction proceeded through bischromene adduct XIII or XIV, respectively, which then generated the chromenopyridine scaffold by reacting with ammonium acetate (Scheme 15). The use of compound **106** afforded **107** in higher yields (54–79% vs. 38–57%). A series of chromenopyridine derivatives **107** were synthesized and tested for their antimicrobial activity for *S. aureus* and *E. coli*, as well as for their antifungal activity against *A. niger* and *C. albicans*, with some compounds, such as **107a**, presenting MIC for *S. aureus* up to 4-fold lower than the standard, ampicillin (Scheme 15).

**Scheme 16:**
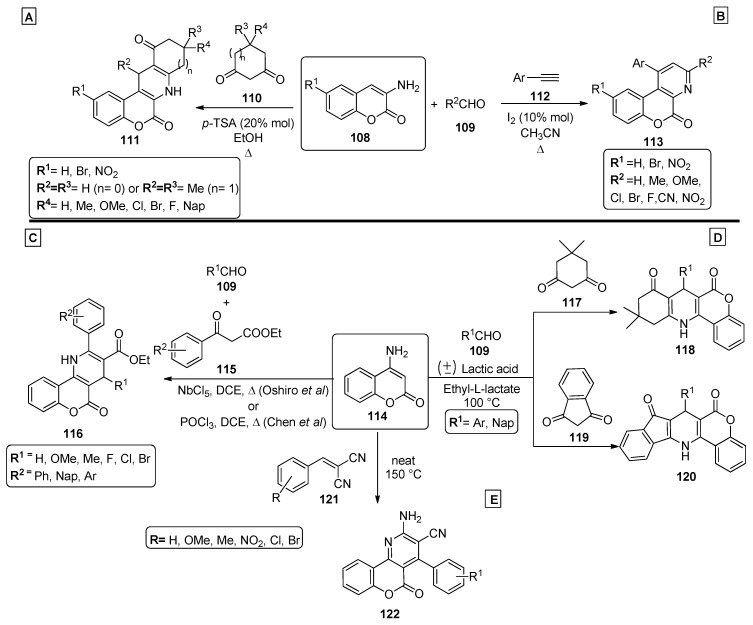
Preparation of chromenopyridines **111**, **113**, **116**, **118**, **120** and **122** (adapted from Refs. [88,89,90,91,92,93]). (**A**,**B**)—Khan et al.; (**C**)—Chen et al., Oshiro et al.; (**D**)—Paul et al.; (**E**)—Motamendi et al.

**Scheme 17:**
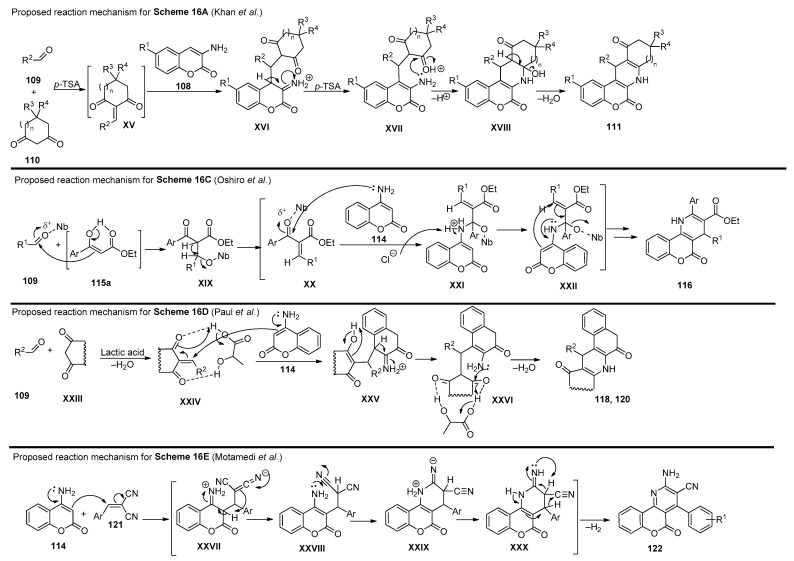
Proposed reaction mechanisms for the formation of compounds **111**, **116**, **118**, **120** and **122** (adapted from Refs. [88,89,90,91,92,93]).

In 2012, Khan and coworkers [88] presented a methodology to obtain chromenopyridine derivatives **111** (72–82% yield) from a three-component reaction involving 3-aminocoumarins **108**, aldehydes **109** and cyclic diketones **110** under reflux in ethanol, catalyzed by p-toluenesulfonic acid (p-TSA) (20% mol) (Scheme 16A). These chromenopyridines were obtained by condensation of **109** with **110**, followed by Michael addition of coumarin **108** via an enamine intermediate. The adduct formed during this process was not isolated and undergoes intramolecular cyclization, followed by dehydration, to afford the desired product **111**. Later, the same author. presented a procedure for the preparation of chromenopyridines **113** [89]. In this work, aminocoumarins **108** were reacted with phenylacetylenes **112**, and the reaction was performed under iodine catalysis and acetonitrile reflux (Scheme 16B). Similarly to their previous work [88], mainly aromatic aldehydes were used. Several exploratory reactions to optimize the reaction conditions were also performed, confirming that a catalyst was necessary for the reaction to occur, and I_2_ (10% mol) was the best catalyst. The proposed mechanism for the formation of chromenopyridines **113** involved an imine intermediate, which in turn undergoes intramolecular cyclization followed by oxidation, to afford the final product. In 2013, Chen et al. [90] prepared chromenopyridines **116** from the reaction of 4-amino-2H-chromen-2-one **114**, aldehydes **106** and ethyl benzoylacetates **115** (Scheme 16C). A series of protic and Lewis acids were tested as catalysts and POCl_3_ was selected as the best catalyst, in a reaction performed under reflux in dichloroethane. Chromenopyridine **116** was oxidized using DDQ under reflux in ethanol. Using this method, 20 derivatives of **116** were prepared in 52–63% yields. Later, Oshiro et al. [91] further explored this procedure, evaluating the performance of other metal-containing Lewis acids, from which niobium chloride (100 mol%) emerged as the most effective catalyst for the three-component reaction of **114**, aldehydes **109** and ethyl benzoylacetate **115**, affording chromenopyridines **116** in 38–58% yields (Scheme 16C). The authors proposed that the Knoevenagel condensation between **109** and the enolic form of ethyl benzoylacetate **115**, as well as the nucleophilic attack of the amine group of **114** to the carbonyl moiety of the Knoevenagel adduct, are facilitated by the niobium catalyst used, which increases electrophilicity in both steps. The work published by Paul et al. [92] also involved aminocoumarins as precursors of chromenopyridines (Scheme 16D). In this work, 4-aminocoumarin **114** was combined with several aldehydes **109**, and with either indane-1,3-dione **117** (to prepare **118**) or cyclohexane-1,3-dione **119** (to prepare **120**), in a reaction performed in ethyl-L-lactate at 100 °C and with (±) lactic acid catalysis (Scheme 16D). These chromenopyridines were isolated in high yields (73–85% for **119**, 83–91% for **120**). The reaction mechanism is similar to that proposed for the reaction in Scheme 16A, starting from the condensation between the diketone compound **117** or **119** and aldehydes **109**, followed by Michael addition of the aminocoumarin **114** and intramolecular cyclization. It was suggested that lactic acid aids the process by interacting with the adduct via hydrogen bonding, facilitating the Michael addition step. Motamendi presented a solvent-free methodology for the synthesis of chromenopyridine derivatives **122**, through the reaction of 4-aminocoumarin **114** with arylidenemalononitriles **121**, at 150 °C [93]. The final product was obtained after flash chromatography purification, with isolated yields of 60–80%. The authors propose that the reaction occurs via Michael addition, where the arylidenemalononitrile acts as the Michael acceptor and the enamine group as the Michael donor, with an intramolecular cyclization step followed by isomerization leading to the chromenopyridine scaffold (Scheme 16E). The respective reaction mechanisms, as proposed by the authors, are presented in Scheme 17.

**Scheme 18:**
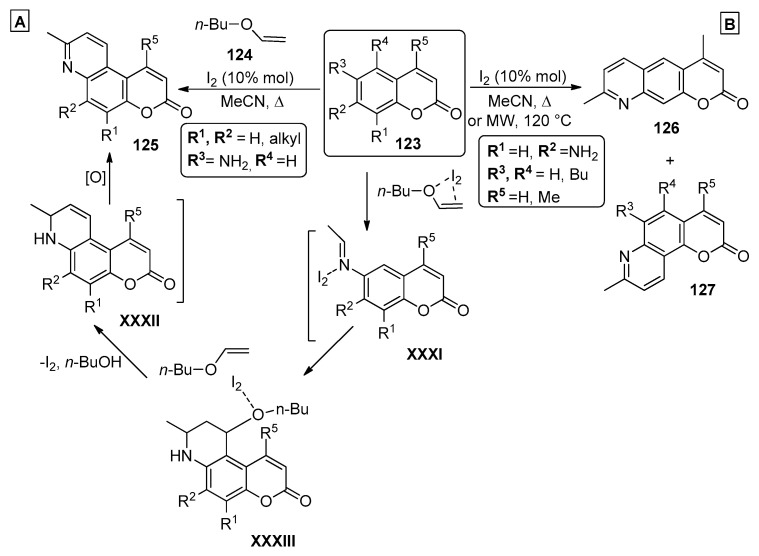
Preparation of chromenopyridines **125–127**, with the mechanism proposed by Symeonidis et al. (adapted from Ref. [94]).

In the past decade, several examples of the use of olefins and aminocoumarins to prepare chromenopyridines have been reported in the literature. One of these procedures was presented by Symeonidis et al. [94], describing an MCR to prepare chromenopyridines. The reaction of 6-amino-7-methylcoumarins **123** (R^3^ = NH_2_) with two equivalents of *n*-butyl vinyl ether **124**, in the presence of a catalytic amount of iodine, in acetonitrile, and under reflux, led to the isolation of compounds **125** in 45–87% yields (Scheme 18A). The reaction starts by a Lewis acid-catalyzed imine formation from one equivalent of both **123** and *n*-butyl vinyl ether **124**, followed by the incorporation of a second equivalent of **124** through a Lewis acid-catalyzed Aza-Diels–Alder reaction. The final product **125** is generated by Lewis acid-catalyzed elimination of *n*-butanol and subsequent oxidation. The authors note that the reaction did not occur in the absence of iodine catalysis, as it is essential in aiding the nucleophilic attack of the amine group to the vinyl ether moiety. An optimization study indicated 10% mol equivalent as the optimal conditions. This methodology was also applied to the preparation of chromenopyridines **126** and **127** from 7-amino-4-methylcoumarins **123** (R^2^ = NH_2_), which could additionally be prepared by performing the reaction under microwave irradiation at 120 °C instead of reflux. Both methods presented similar results, although none of the proposed methods were regioselective, affording the chromenopyridines **127** as secondary products (Scheme 18B).

**Scheme 19:**
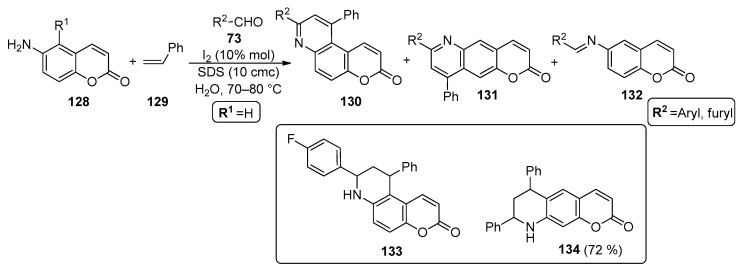
Preparation of chromenopyridines **130**–**132**, with the chemical structures of the side products **133** (identified in a mixture with **130** and **132**) and **134** highlighted (adapted from Ref. [95]).

Iodine catalysis was also used to prepare chromenopyridines **130**–**132** in an MCR between 6-aminocoumarins **128**, aromatic aldehydes **73** and styrene **129** (Scheme 19). Ganguly and Chandra [95] reported that the reaction occurs in an aqueous medium, using SDS as a micellar auxiliar. The optimized reaction conditions involved the use of coumarin/aldehyde/styrene in a 1:1.1:4 ratio, in aqueous SDS (10 cmc) and I_2_ (10% mol), at 70–80 °C. It was mentioned that SDS behaves as an activator of iodine, important in the formation of the intermediate Schiff base from the reaction of **128** with the aromatic aldehyde **73**. These conditions were applied to a wide variety of aromatic aldehydes, which afforded mixtures of **130**, **131** and **132** in variable yields (25–72% for **130**, 10–40% for **131** and 7–40% for **132**). Nevertheless, compound **130** was always isolated as the major product. Compounds **131** and **132** were also isolated when aldehydes **73** were used. In the reaction of 6-aminocoumarin and *p*-iodobenzaldehyde, the non-oxidized form was isolated (**133**) and in the reaction of 6-amino-5-bromocoumarin with benzaldehyde, a product incorporating two equivalents of styrene was isolated (**134**) (Scheme 19).

### 2.5. Miscellaneous

**Scheme 20:**
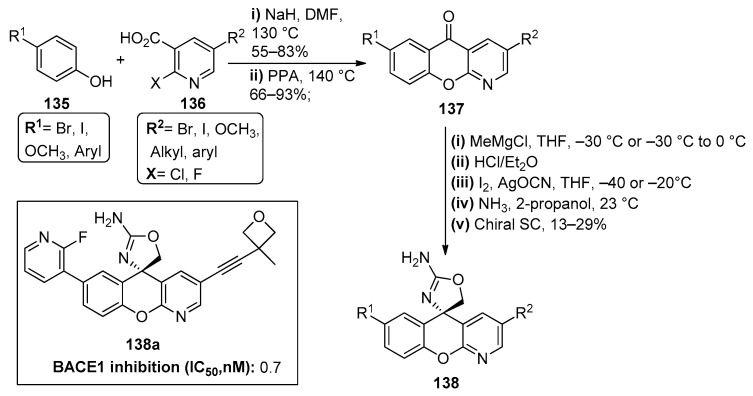
Preparation of chromenopyridine scaffold **138**, used for the preparation of 16 derivatives, as proposed by Dineen et al., with the respective biological activity (BACE1 inhibition, µg/mL) highlighted for compound **138a** (adapted from Refs. [96,97,98,99]).

The literature also reports the formation of the chromenopyridine moiety through coupling reactions. Several publications [96,97,98] on the development of new β-site amyloid precursor protein cleaving enzyme (BACE1) inhibitors reported the synthesis of a set of xanthene derivatives. However, after a series of molecular optimization studies, it was observed that the best results for balancing BACE1 potency, P-glycoprotein-mediated efflux and human *Ether-à-go-go*-Related Gene (hERG) binding affinity could be achieved using a chromenopyridine scaffold [99]. The chromenopyridines **137** prepared in this study were generated by coupling a selection of *p*-substituted phenols 1**35** (R^1^ = Br, I, OCH_3_) with 2,5-substituted nicotinic acids **136**, using NaH as catalyst, followed by ring closure of the adduct by neat polyphosphoric acid at 140 °C (Scheme 20). The enantioselective preparation of chromenopyridines **138** with an aminooxazole moiety was obtained by a three-step methodology: (i) addition of a Grignard reagent (CH_3_MgCl) to **137** and subsequent treatment with HCl in diethyl ether to afford an unstable *exo*-olefin; (ii) treatment of the olefin with the in situ generated iodine isocyanate at −20 °C; (iii) treatment with a solution of ammonia in 2-propanol at 23 °C, to generate a racemic mixture of the 2-aminooxazole chromenopyridine derivative. The pure (*S*)-enantiomer of compound **138** was obtained after chiral supercritical fluid (SCF) separation, in 13–29% yields, depending on the substituents present in the product. These chromenopyridines were then used in a variety of subsequent reactions to prepare a library of compounds with different substituents R^1^ and R^2^. From this library, compound **138a** is highlighted, with a promising inhibitory capacity of BACE1, among other important physicochemical properties (Scheme 20).

## 3. Conclusions

Chromono- and chromenopyridines combine two of the most widely recognized biologically active structural motifs, the chromene and pyridine nucleus. Bibliographical information from the years of 2010–2023 on different strategies for chromenopyridine synthesis, as well as their promising associated biological properties, have reinforced what was observed until 2010: there is a strong interest in the scientific community concerning this type of compound and its biological use. This interest has led not only to improvements on already known synthetic methods, but also to developments of new insightful methods. Furthermore, this review contributes to a thorough compilation of those discoveries, to fuel further advancements on the development of novel bioactive chromenopyridines with the potential for clinical translation.
